# Weak electric-field detection with sub-1 Hz resolution at radio frequencies using a Rydberg atom-based mixer

**DOI:** 10.1063/1.5095633

**Published:** 2019

**Authors:** Joshua A. Gordon, Matthew T. Simons, Abdulaziz H. Haddab, Christopher L. Holloway

**Affiliations:** 1 National Institute of Standards and Technology (NIST), RF Technology Division, U.S. Department of Commerce, Boulder Laboratories, Boulder, Colorado 80305, USA; 2 Department of Physics, University of Colorado, Boulder, Colorado 80302, USA; 3 Department of Electrical Engineering, University of Colorado, Boulder, Colorado 80302, USA

## Abstract

Rydberg atoms have been used for measuring radio-frequency (RF) electric (E)-fields due to their strong dipole moments over the frequency range of 500 MHz-1 THz. For this, electromagnetically induced transparency (EIT) within the Autler-Townes (AT) regime is used such that the detected E-field is proportional to AT splitting. However, for weak E-fields AT peak separation becomes unresolvable thus limiting the minimum detectable E-field. Here, we demonstrate using the Rydberg atoms as an RF mixer for weak E-field detection well below the AT regime with frequency discrimination better than 1 Hz resolution. A heterodyne detection scenario with two E-fields incident on a vapor cell filled with cesium atoms is used. One E-field at 19.626000 GHz drives the $34D_{5/2}\to 35P_{3/2}$ Rydberg transition and acts as a local oscillator (LO) and a second signal E-field (Sig) of interest is at 19.626090 GHz. In the presence of the LO, the Rydberg atoms naturally down convert the Sig field to a 90 kHz intermediate frequency (IF) signal. This IF signal manifests as an oscillation in the probe laser intensity through the Rydberg vapor and is easily detected with a photodiode and lock-in amplifier. In the configuration used here, E-field strength down to ≈46μV/m±2μV/m were detected with a sensitivity of $\approx 79\mu {\text{Vm}}^{-1}{\text{Hz}}^{-1/2}$. Furthermore, neighboring fields 0.1 Hz away and equal in strength to Sig could be discriminated without any leakage into the lock-in signal. For signals 1 Hz away and as high as +60 dB above Sig, leakage into the lock-in signal could be kept below −3 dB.

Rydberg atoms^1^ have been demonstrated as quantum sensors for electric(E)-field metrology over the radio frequency (RF) range of approximately 500 MHz-1 THz, and have properties not found in classical E-field sensors, such as sub RF-wavelength size,^2–5^ self calibration,^6,7^ and system international (SI) traceability to Plank’s constant.^8^ Electromagneticlly induced transparency^9–11^(EIT), and Autler-Townes (AT) splitting^12^ used to realize the Rydberg atom E-field sensor, reduce an RF E-field measurement to an optical frequency measurement. Progress has been made using Rydberg atoms to characterize classical properties of RF E-fields including magnitude,^6,7,13,14^ polarization,^15^ phase^16^ and, power.^17^ More recently the concept of the Rydberg E-field sensor has been expanded in the form of the “Rydberg Atom Receiver” and “Rydberg Atom Radio”^5,18–22^ which have been used to detect time varying fields of common modulation schemes such as QPSK, AM, and FM.

The detection of weak RF fields (i.e. below 1 mV/m) is important for practical applications if the Rydberg atom RF field sensor is to compete with traditional circuit based sensors. Several techniques have been proposed to improve signal to noise levels for weak RF E-field measurements such as, using optical cavities^23^ to narrow the EIT line width and improve AT splitting resolution, homodyne detection with a Mach Zehnder interferometer^24^ and frequency modulated spectroscopy.^25^ Previously, we reported on the Rydberg atom mixer^16^ for determining the phase of an RF field. Here, in a continuation of exploring the Rydberg atom mixer, we show how this mixer effect can also be applied for the detection of weak RF fields that are well below AT splitting with the added benefit of isolation of signals at adjacent frequencies, and frequency selectivity of ∼ 10^8^ better than that provided by the Rydberg transition alone. Using the Rydberg atom mixer we demonstrate a weakest detectable field of ≈46μV/m±2μV/m with a sensitivity of $\approx 79\mu {\text{Vm}}^{-1}{\text{Hz}}^{-1/2}$ without the need for cavities or inteferometers with better than ∼ 1 Hz resolution.

The setup for this work is shown in Fig. 1. Rydberg atoms are produced using a 75 mm×25 mm (Length×Diameter) cylindrical glass atomic vapor cell filled with cesium (^133^Cs) atoms. A probe laser tuned to the the D2 transition wavelength of $\lambda_{p}=852\;\text{nm}$ excites the ^133^Cs from the ground state to the first excited state $\left(6S_{1/2}\to 6P_{3/2}\right)$. A counter propagating coupling laser is tuned to $\lambda_{c}=511.148\text{nm}$, and further excites the ^133^Cs atoms to the Rydberg state $34D_{5/2}$ thus producing a transparency region in the probe laser spectrum. The probe laser beam has a full-width half-maximum (FWHM) of 425 *μ*m and a power of 49 *μ*m, the coupling laser has a FWHM of 620 *μ*m and a power of 60.6 mW. Under these conditions an incident RF field operating near the frequency of 19.626 GHz drives the $34D_{5/2}\to 35P_{3/2}$ transition. With the probe laser frequency fixed on resonance with the D2 transition, the transmission through the vapor cell is in general reduced when in the presence of the applied RF field. For appreciable field strengths the atoms are driven to the Autler-Towns regime^12^ which splits the observed EIT peak in the probe laser transmission spectrum. The frequency separation $\text{Δ}f_{AT}$ of the two AT peaks is given^6,14^ by,

(1) $$\text{Δ}f_{AT}=\frac{\lambda_{c}}{\lambda_{p}}\frac{E_{RF℘RF}}{2\pi \hbar }$$

Where ℘RF is the dipole matrix element of the RF Rydberg transition and ħ is Plank’s constant. The dipole moment for the resonant RF transition is $℘=723.3739ea_{0}$ (which includes a radial part of $1476.6048ea_{0}$ and an angular part of 0.48989, which correspond to co-linear polarized optical and RF fields, where e is the elementary charge; $a_{0}=0.529177\times 10^{-10}\text{m}$ and is the Bohr radius). AT splitting as a method for E-field sensing becomes less effective for E-fields too weak to cause resolvable AT peak separation. The work described below overcomes this weak E-field limitation through the Rydberg atom mixer effect with the added benefit of narrow band frequency selection and tuning. Here, we define the minimum detectable RF field capable of being detected with AT splitting as that which causes an AT peak separation equivalent to the EIT line width ${\text{Γ}}_{EIT}$. From (1) this is,

(2) $$E_{AT}=\frac{\lambda_{p}}{\lambda_{c}}\frac{2\pi \hbar {\text{Γ}}_{EIT}}{{℘}_{RF}}.$$

As determined from the EIT spectrum shown in Fig. 2, ${\text{Γ}}_{EIT}\approx 4\;\text{MHz}$ and $E_{AT}=0.72\;\text{V}/\text{m}$ for the above mentioned Rydberg states.

A schematic of the Rydberg atom mixer^16^ is shown in Fig. 3. A heterodyne detection scenario is used where two different RF fields are incident on the vapor cell, $E_{1}=E_{LO}\cos\left(\omega_{LO}t+\phi_{LO}\right)$, and $E_{2}=E_{\text{Sig}}\cos\left(\omega_{\text{Sig}}t+\phi_{\text{Sig}}\right)$. One is tuned to $f_{LO}=\omega_{LO}/2\pi =19.626000\;\text{GHz}$ such that it is on resonance with $34D_{5/2}\to 35P_{3/2}$ Rydberg transition. This field acts as a local oscillator (LO). The second field $E_{2}$ is the signal field (Sig) that is to be sensed and is tuned to $f_{\text{Sig}}=\omega_{\text{Sig}}/2\pi =19.626090\;\text{GHz}$ such that it is detuned by +90 kHz from the LO field. Here, we explore the case when both $E_{1}$ and $E_{2}$ are co-polarized and considered weak where $E_{1}\approx E_{AT}$ and $E_{2}\leq E_{AT}$.

The interference occurring from the superposition of these fields results in a high frequency component $E_{res}$ and low frequency component $E_{\text{mod}}$. With $\bar{\omega }=\left(\omega_{LO}+\omega_{\text{Sig}}\right)/2,\text{Δ}\omega =\omega_{LO}-\omega_{\text{Sig}}$, and $\text{Δ}\phi =\phi_{LO}-\phi_{Sig}$, for small relative detuning where $\text{Δ}\omega /\bar{\omega }\ll 1$ the total field at the atoms $E_{atoms}$ can be shown to be,

(3) $$E_{\text{atoms}}=E_{1}+E_{2}$$

(4) $$=\cos\left(\omega_{LO}t+\phi_{LO}\right)\sqrt{E_{LO}^{2}+E_{Sig}^{2}+2E_{LO}E_{Sig}\cos(\text{Δ}\omega t+\text{Δ}\phi )}$$

(5) $$=E_{\text{res}}\times E_{\text{mod}}.$$

Where $E_{\text{res}}$ oscillates at $\omega_{LO}$ and $E_{\text{mod}}$ oscillates at Δω. The magnitude of the total field is given by,

(6) $$\left|E_{atoms}\right|=\sqrt{E_{LO}^{2}+E_{\text{Sig}}^{2}+2E_{LO}E_{\text{Sig}}\cos(\text{Δ}\omega t+\text{Δ}\phi )}.$$

For weak fields where $E_{Sig}\ll E_{LO}$, (6) becomes,

(7) $$\approx E_{LO}+E_{Sig}\cos(\text{Δ}\omega t+\text{Δ}\phi ).$$

The Rydberg atoms have a naturally different response to $E_{res}$ and $E_{mod}$. Since $E_{res}$ oscillates at $\omega_{LO}$ it is resonant with the Rydberg transition, where as $E_{mod}$ oscillates at a frequency that is well below the Rydberg transition frequency and results in a modulation of the EIT spectrum and thus the probe laser intensity on the photodiode (see Fig 1). The effect being the down conversion of the incident field $E_{2}$ from the base band RF frequency of $\omega_{\text{Sig}}$ to an intermediate frequency (IF) of $f_{IF}=\text{Δ}\omega /(2\pi )$ (see Fig. 3),

(8) $$E_{IF}=E_{LO}+E_{Sig}\cos(\text{Δ}\omega t+\text{Δ}\phi ).$$

In this case the probe laser intensity on the photodiode varies at $f_{IF}=90\;\text{kHz}$. A detectable IF signal is produced even for $E_{sig}$ well below $E_{AT}$. Fig. 4 shows time domain plots of the IF signal out of the photodiode for various $E_{sig}$ levels. The 90 kHz modulation is easily seen as is the changing modulation amplitudes following the behavior of (8). For the final stage of detection the output of the photodiode is passed to a lock-in amplifier with a reference set equal to the IF frequency, $f_{REF}=f_{IF}$. The lock-in output voltage $\left(V_{LI}\right)$ is thus proportional to weak field, $V_{LI}\propto E_{Sig}$.

Two identical source antennas (Narda 638 horns were used, however mentioning this product does not imply an endorsement by NIST, but only serves to clarify the equipment used) were used to produce $E_{LO}$ and $E_{Sig}$ fields. The antennas were placed 385 mm from the ^133^Cs vapor cell such that they were beyond the $2{\text{a}}^{2}/\lambda_{RF}=305\;\text{mm}$ far field distance.^26^ Where a=48.28mm is taken as the diagonal length of the antenna aperture and $\lambda_{RF}=15.286\;\text{mm}$. Two separate RF signal generators synced via a 10 MHz reference were used to feed the two antennas at frequencies of $f_{LO}=19.62600\;\text{GHz}$, and $f_{\text{Sig}}=19.626090\;\text{GHz}$. A calibrated power meter and vector network analyzer were used to account for cable loss from the RF signal generator and horn reflection coefficient and to determine the RF power at the horn antennas $P_{RF}$. For powers down to −70 dBm the power meter was used. For weak field generation $P_{RF}$ was <−70 dBm and thus well below the dynamic range of an RF power meter. To overcome this, the signal generator was operated within the range of the power meter from +10 to −60 dBm and additional calibrated attenators were added providing up to − 111 dB of additional loss. With this configuration accurate control of power levels could be achieved down to ≈ −180 dBm.

To accurately determine the E-field within the vapor cell for low RF powers into the horns, AT splitting was used to calibrate and correct errors imparted on the E-field due to the presence of the vapor cell. As has been shown in Refs. 2, 27, and 28 for an RF field incident on a vapor cell, scattering off of the glass walls can cause internal resonances and alter the E-field amplitude inside the vapor cell from that which would exist given the vapor cell were not there. The E-field at the horn-to-laser beam distance R=385mm was calculated/using^26,29^ the far-field formula $E_{FF}=\sqrt{59.9585}\sqrt{P_{RF}G}/R$ where the antenna gain is G=15.55dB±0.4dB. For a given distance R and RF frequency there is a fixed ratio of the E-field inside the vapor cell $E_{cell}$ to the E-field in the absence of vapor cell $E_{FF}$. This is given by the cell factor $C_{f}=E_{cell}/E_{FF}$. Calibration data for $E_{cell}$ was determined from the conventional AT splitting technique (1) for a range of $P_{RF}$ strong enough to cause AT splitting. Cell factor calibration data comparing $E_{cell}$ and $E_{FF}$ is shown in Fig. 5. Given the uncertainty in G, power meter, and operating within the linear response^30^ of the AT regime (1), weak E-fields detected by the Rydberg mixer could be known for a given $P_{RF}$ to within an estimated uncertainty of ± % 5. For the configuration used here $C_{f}=0.90$ and thus for a given $P_{RF}$,

(9) $$E_{Cell}=\frac{0.90\sqrt{59.9585}\sqrt{P_{RF}G}}{R}$$

Weak E-field data (blue squares) are plotted in Fig. 6 for lock-in amplifier output voltage-vs- $-\sqrt{P_{RF}}$ along with the corresponding E-field strength. For these data a 3 s time constant (bandwidth of $f_{c}=0.33\;\text{Hz}$) and 24 dB/octave low pass filter slope was used. As $P_{RF}$ approaches powers < −100 dBm the lock-in signal approaches the noise floor which shows up by the flattening out of the data curve. To estimate the repeatably of the measurement, 3 sets of data were acquired for each E-field level. This showed a 5 % variation. Also shown in Fig. 6 are the higher E-fields that were used for cell factor calibration and acquired from AT splitting. These data (red circles) follow the linear behavior predicted by equations (1) and (9). The weak E-field data remains linear up until $E_{AT}$ is reached. The cross over between the weak field regime and AT regime shows up as a roll off of the weak field data near $E_{AT}$. This roll off is due to the EIT peak center frequency shifting away from the probe laser frequency as AT splitting begins to take place. The weakest detectable E-field is taken as the value at where the lock-in voltage curves to the noise floor. This corresponds to ≈46μV/m and a sensitivity of $\approx 79\mu {\text{Vm}}^{-1}{\text{Hz}}^{-1/2}$.

Another aspect of the Rydberg mixer is its ability to isolate and discriminate between signals of differing RF frequencies with a frequency resolution orders of magnitude finer than the response bandwidth of the Rydberg transition. As was shown in Ref. 31, through the generalized Rabi frequency, RF E-fields that are off-resonance with the Rydberg transition will still affect the EIT spectrum over a large continuum of frequencies of hundreds of MHz. For an RF frequency detuning of $\delta_{RF}$, and on-resonance Rabi frequency of ${\text{Ω}}_{o}$, the generalized Rabi frequency becomes, ${\text{Ω}}^{\prime }=\sqrt{{\text{Ω}}_{o}^{2}+\delta_{RF}^{2}}$. For example in the AT regime, splitting will still occur for off-resonance E-fields for a large range of $\delta_{RF}$, where now the splitting $\text{Δ}f_{AT}\to {\text{Ω}}^{\prime }/(2\pi )$. As such, discriminating between E-fields of different RF frequencies through purely observing the EIT spectrum becomes difficult and ambiguous. The Rydberg atom mixer provides a means to overcome this so that E-fields differing in frequency by as little as 1 Hz can be discriminated. For this, the lock-in amplifier is tuned to the desired IF frequency corresponding to the desired down converted RF frequency. Simply tuning $f_{REF}$ allows for signals at different RF frequencies to be discriminated and isolated.

We demonstrate this and examine the leakage in the lock-in signal for E-fields at neighboring frequencies and various strengths relative to the “in-tune” E-field. First, an in-tune IF signal was produced where the RF signal generator power was set to roughly middle of range at $P_{RF}=-40\;\text{dBm}$ and $f_{IF}=90\;\text{kHz}$. This signal we denote as $E_{o}=181\;\mu \text{V}/\text{m}$. The lock-in reference was also tuned to $f_{REF}=90\;\text{kHz}$, and a time constant of 3 s, giving a cut off frequency of $f_{c}=0.33\;\text{Hz}$. Three other signals denoted as $E_{\text{Δ}f}$ that were out of tune by Δf=0.1Hz, Δf=1Hz, Δf=10 were also produced. For these three signals $P_{RF}$ was then varied such that $E_{\text{Δ}f}/E_{o}$ ranged from 0 dB to greater than 60 dB. Fig. 7 shows a plot of the lock-in output for the three detuned signals normalized to the level produced by $E_{o}$. The lock-in noise floor is depicted as well. As can be seen there is a range of relative strengths for each detuned signal where the lock-in signal is at the noise floor and then rises up to equal the level of $E_{o}$. All three detunings show maximum isolation when equal to $E_{\text{Δ}f}/E_{o}=0\;\text{dB}$. Where even for sub-Hz detuning of Δf=0.1Hz, $E_{\text{Δ}f}$ does not rise above the noise floor. The isolation threshold in dB for each detuning is taken for the value of $E_{\text{Δ}f}/E_{o}$ that crosses −3 dB level of the lock-in signal. Isolation degrades more quickly for smaller detunings for $E_{\text{Δ}f}/E_{o}>1$. For a detuning of Δf=1Hz the −3 dB crossing happens for $E_{\text{Δ}f}/E_{o}\approx 60\;\text{dB}$.

This work shows E-field strengths −84 dB below the AT limit $E_{AT}$ can be detected using the Rydberg atom mixer.^16^ Furthermore, the Rydberg atom mixer allows specific RF frequencies to be selected, isolated and rejected with resolution better than 1 Hz. This is a ∼ 10^8^ improvement in RF frequency resolution over that provided by the frequency bandwidth^31^ of the Rydberg transition alone. These attributes along with the ability to measure phase,^16^ and polarization^15^ allow for the development of a quantum-based sensor to fully characterize the RF E-field in one compact vapor cell.

## Figures and Tables

**FIG. 1.**
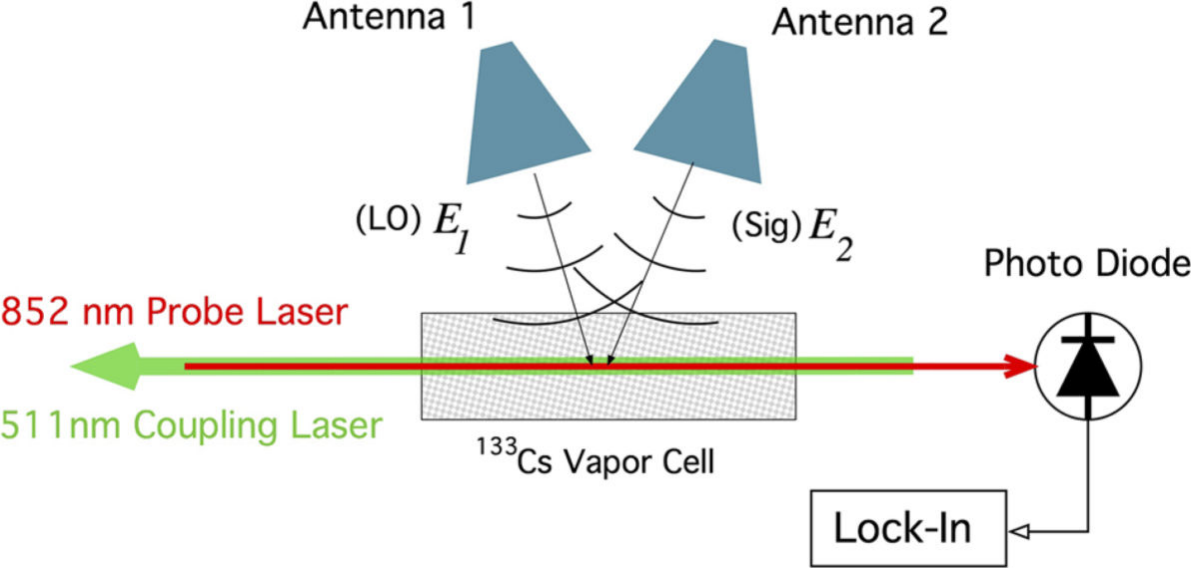
Diagram of experimental setup. E-field $E_{1}$ acting as a local oscillator (LO) is produced by Antenna 1 while Signal (Sig) E-field $E_{2}$ is produced by Antenna 2. Both fields are superposed along with the probe and coupling lasers at the ^133^Cs vapor cell. Probe laser is incident on the photodiode with output passed to a lock-in amplifier.

**FIG. 2.**
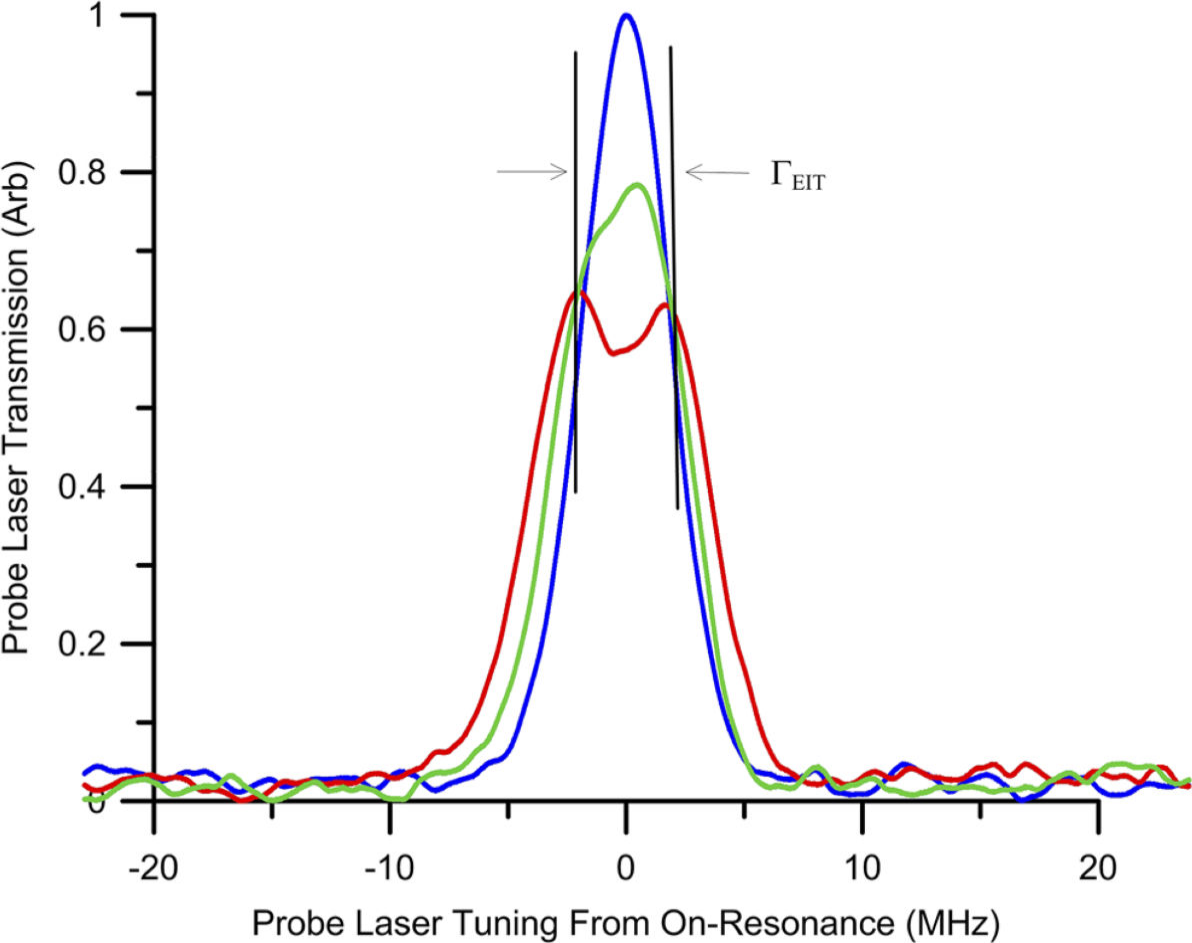
Probe laser spectrum plots showing the transition into the AT regimes. (Blue) no RF field where E=0V/m, (Green) $E<E_{AT}$, (Red) $E=E_{AT}$ the EIT peak just begins to split into two resolvable peaks separated by ${\text{Γ}}_{EIT}$.

**FIG. 3.**
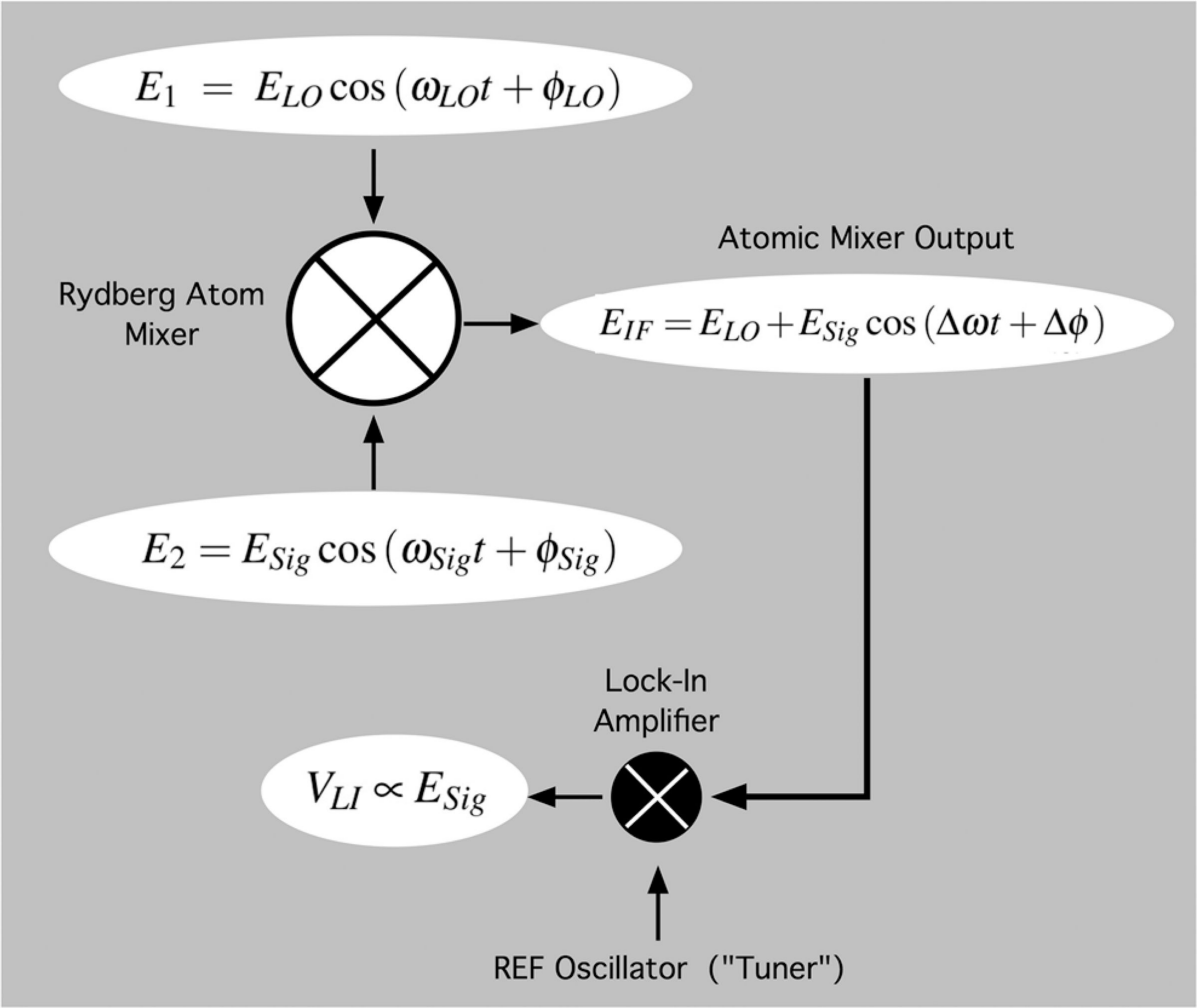
Flow chart showing the application of the Rydberg atom mixer to weak field detection. Inputs Local Oscillator (LO) field $E_{1}$ and Signal (Sig) field $E_{2}$, produce an IF field $E_{IF}$ output which is detected by a lock-in amplifier producing a voltage $V_{LI}$ proportional to $E_{Sig}$. The reference oscillator (REF Oscillator) for the lock-in is set to $f_{REF}=f_{IF}$ and allows narrow band ≲1Hz selective tuning to isolate Sig fields having a range of differing frequencies relative to the LO frequency.

**FIG. 4.**
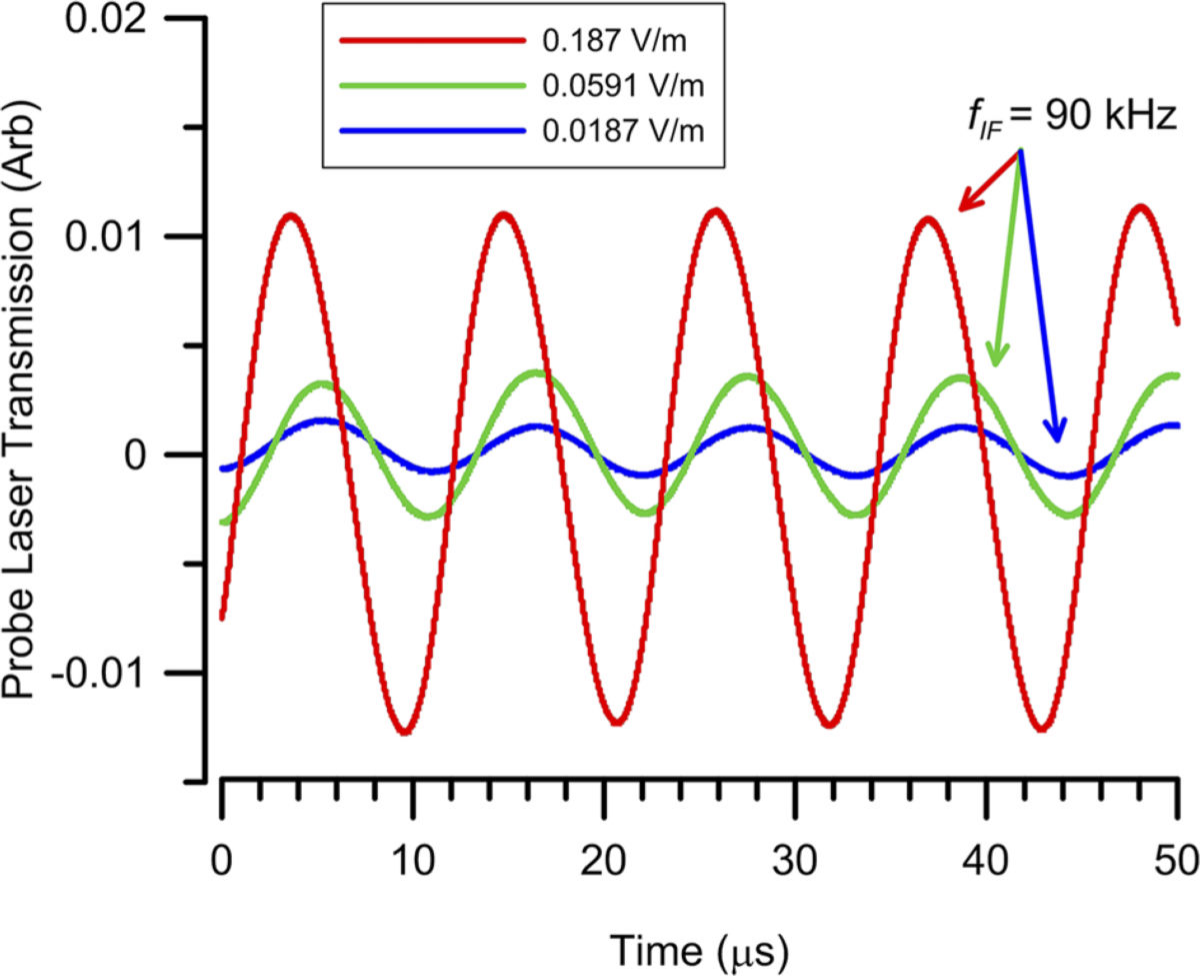
Time domain plots of the IF signal from the photodiode for $E_{Sig}=0.187\;\text{V}/\text{m}$, 0.0591 V/m, 0.0187 V/m.

**FIG. 5.**
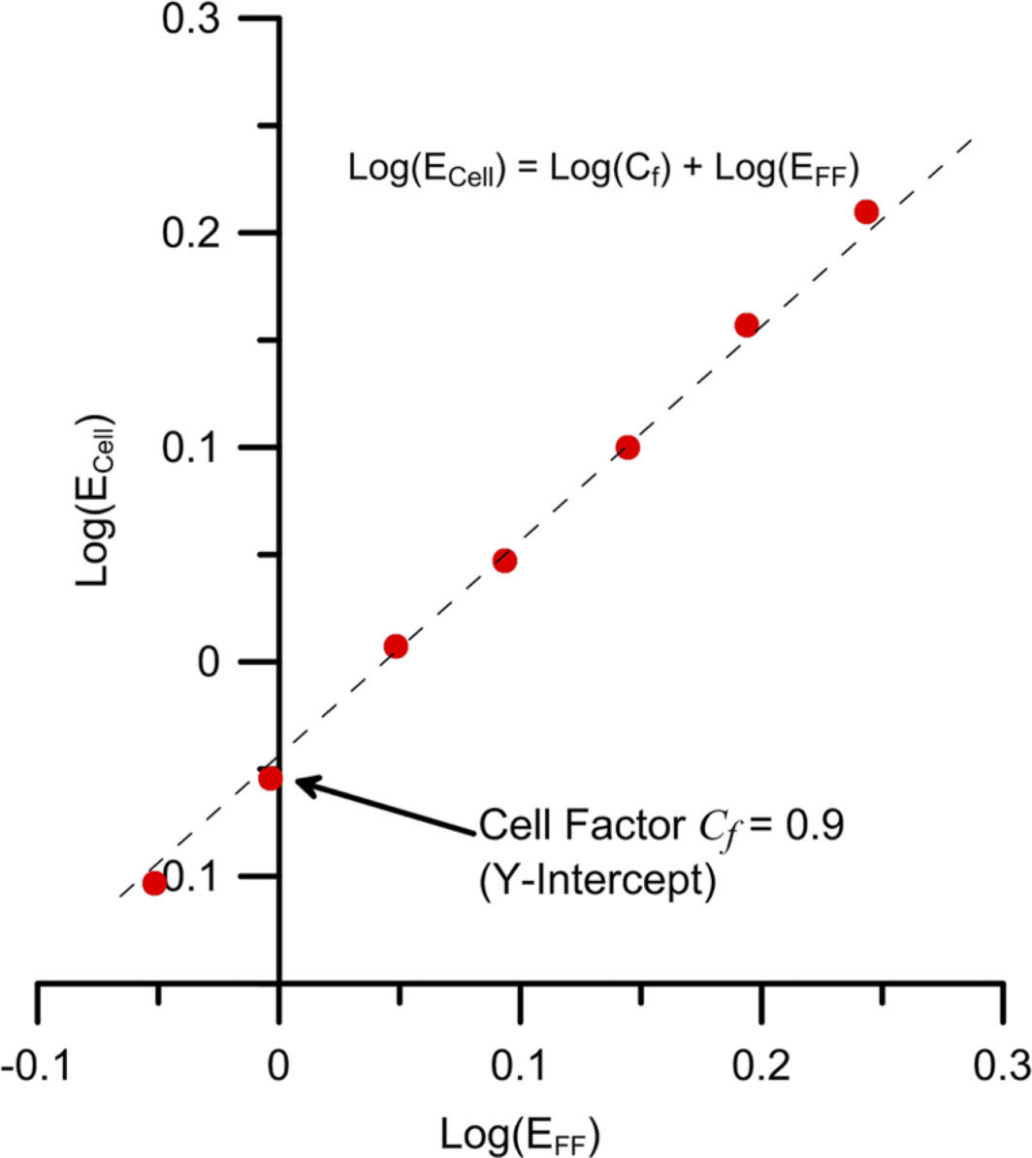
Plot of $Log\left(E_{Cell}\right)-v.s.-Log\left(E_{FF}\right)$. $C_{f}$ is given by the Y-intercept.

**FIG. 6.**
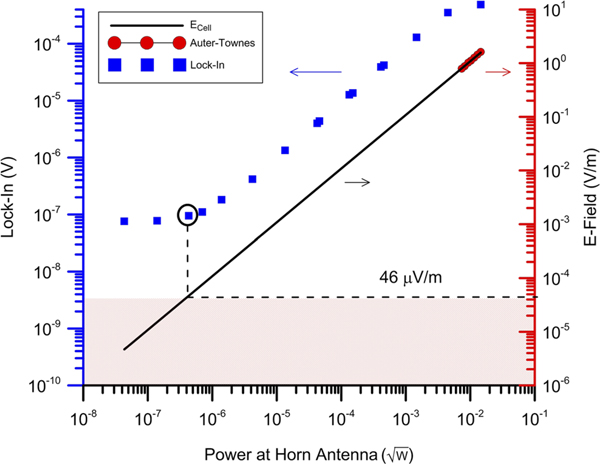
Detection plot for weak fields. (blue, left-axis) lock-in signal, (red, right-axis) AT splitting, and (line, right-axis) $E_{C\text{ell}}$ as a function of $\sqrt{P_{RF}}$. (dashed-line) Lock-in signal corresponding to lowest detectable E-field of 46μV/m.

**FIG. 7.**
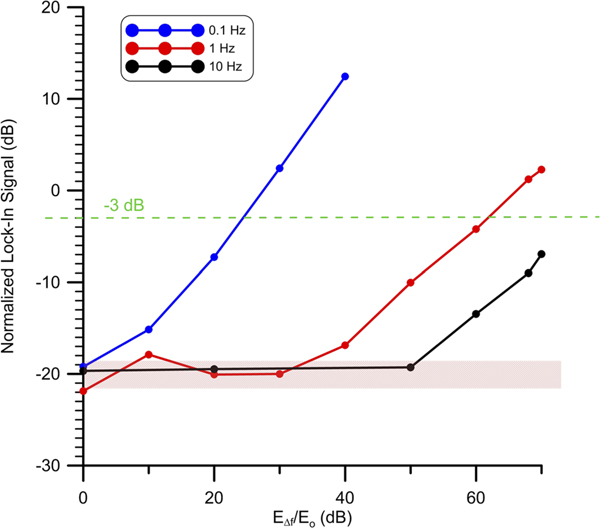
Isolation of neighboring signals for various E-field strengths relative to $E_{0}$ and for Δf=0.1Hz, 1 Hz, and 10 Hz. Lock-in signal is normalized to that produced by $E_{0}=181\;\mu \text{V}/\text{m}$. Signals below − 3 dB level are considered to be isolated. Noise floor around − 20 dB is shown by red region.
